# 

**Published:** 1888-02

**Authors:** 


					CONTENTS:
Article I.-
-On the Curability of Pulpless and Abscessed Teeth ;
M. D.
433
D. D. S.
447
455
45?
.OU|S
p the
Standpoint of Physiological Chemistry.
By L. G. Noel, M. D., D. D. S
404
VII.-
Extracts trom Lectures on Operative uentai Surgery.
By William St. George Elliott, M. D., D. D. S .
469
BIBLIOGRAPHICAL.
Dental Chemistry?The Dentist's Manual of Special Chemistry.
474
Memoirs of a Southern Planter
475
Irregularities ol the leeth and their ireatment.
47?
Vick's Floral Guide
476
Nitrous Oxide?Its Properties?Methods ot Administration and kttects.
477
The Physician's Visiting List, 1888
477
Pearson's Dentists Appointment Book
477
Transactions of the American Dental Association.
477
MONTHLY SUMMARY.
Pure Mercury
478
Chinese Anaesthetic
47?
The Control of Haemorrhage
479
A Receipt for Mending Broken Marble
479
Mastication
4?o
Hours ot rate
4?o
Difference Between Creosote and Carbolic Acid
4?o
Mercurial Paralysis
48q

				

